# Clinical improvement of photophobia with galcanezumab in episodic and chronic migraine

**DOI:** 10.3389/fneur.2026.1792472

**Published:** 2026-06-01

**Authors:** Elif Ilgaz Aydınlar, Pınar Yalınay Dikmen, Aysenur Sahin, Kerem Birısık

**Affiliations:** 1 Department of Neurology, School of Medicine, Acibadem University, Istanbul, Türkiye; 2 School of Medicine, Acibadem University, Istanbul, Türkiye

**Keywords:** CGRP monoclonal antibody, galcanezumab, migraine, photophobia, UPSIS-12

## Abstract

**Objective:**

This study aimed to evaluate changes in photophobia using the Turkish version of Utah Photophobia Symptom Impact Scale–12 (UPSIS-12) in patients with episodic (EM) and chronic migraine (CM) treated with galcanezumab, and to examine the relationship between photophobia, migraine outcomes, disability, and treatment response.

**Background:**

Photophobia is one of the most bothersome symptoms (MBS) of migraine and may persist during both ictal and interictal periods, leading to substantial impairment in daily functioning. Despite the clear clinical relevance of photophobia, the specific effects of galcanezumab, a CGRP monoclonal antibody, on photophobia have not yet been fully elucidated.

**Methods:**

This retrospective study, included patients with EM and CM treated with galcanezumab (240 mg loading dose followed by monthly 120 mg) for 3 months. Patients completed headache diaries and validated patient-reported outcome measures (PROMs) at baseline and monthly follow-up visits. Non-parametric tests, correlation analyses, and multivariable linear regression were performed.

**Results:**

A total of 77 patients were enrolled, of whom 47 (89.3% female; mean age 39.5 ± 10.6 years) completed all study visits and were included in the final analysis. Over the study period, monthly headache days (MHDs) decreased from a median of 15 to 4 days by month 3 (*p* < 0.001), with 74.5% of patients classified as responders (≥50% reduction). UPSIS-12 scores declined significant (median 22.6%; *p* < 0.001). Although baseline UPSIS-12 scores were similar between responders and non-responders, responders demonstrated a notable reduction in ictal photophobia (*p* = 0.010). Significant improvements were also observed in ictal phonophobia, osmophobia, and cutaneous allodynia, whereas interictal sensory symptoms remained largely unchanged. In addition, headache intensity, attack duration, acute medication use, disability (HIT-6, MIDAS), and comorbid depression (BDI) and anxiety (BAI) scores all improved. Treatment was well tolerated, with no serious adverse events reported.

**Conclusion:**

Galcanezumab was associated with clinically important reduction not only in migraine frequency, severity, disability, and psychological comorbidities, but also in ictal photophobia, highlighting its responsiveness to CGRP-targeted preventive therapy. These findings suggest that photophobia as a potentially modifiable outcome that warrants systematically evaluation in future studies, with larger cohorts and long-term follow-up.

## 1 Introduction

Photophobia, defined as an abnormal sensitivity or intolerance to light, is a core sensory feature of migraine with a significant impact across the disease spectrum (1). It is included in the International Classification of Headache Disorders, 3rd edition (ICHD-3) as a key accompanying symptom for the clinical diagnosis of migraine (2). Beyond its diagnostic role, photophobia has consistently been identified as MBS. In the MAST study, 49.1% of participants (2,967 of 6,045) reported photophobia as their primary migraine-related complaint, exceeding the proportions reported for nausea (28.1%) and phonophobia (22.8%) (1). Consistent with these findings, the Turkish real-world galcanezumab study similarly identified photophobia as the most frequently reported migraine-related MBS, affecting 40.3% of patients prior to treatment (3). Importantly, photophobia is not limited to ictal periods but may persist interictally, where it substantially impairs quality of life by restricting patients’ ability to work, study, and engage in social and leisure activities (4).

Despite being extensively studied, photophobia has rarely been documented and evaluated in detail, primarily due to the limited availability of standardized, validated tools. As a result, evidence regarding the effectiveness of treatments in reducing photophobia is still limited. UPSIS-12 was designed to fill this gap, offering a validated patient-reported outcome measure that allows for a more precise evaluation of both ictal while placing particular emphasis on the often-overlooked interictal photophobia and its impact on daily functioning in patients with migraine (5, 6). Ictal photophobia, when it occurs during migraine attacks, may also be present on headache-free days, in which case it is referred to as interictal photophobia (7).

Recent advances in migraine pathophysiology have identified CGRP as a critical neuropeptide in the initiation and maintenance of migraine attacks. CGRP is released from trigeminal nerve endings during migraine episodes, leading to vasodilation, neurogenic inflammation, and activation of the trigeminovascular system, which together contribute to pain transmission and central sensitization (8, 9). Elevated interictal CGRP levels have been demonstrated in migraine patients compared with healthy controls, supporting its role as both a biomarker and therapeutic target (10). Experimental studies have showed that intravenous CGRP can induce light-aversive behavior and cephalic allodynia, supporting its role in migraine-related photophobia (11). CGRP likely acts outside the blood–brain barrier in migraine and photophobia, yet it can indirectly modulate central nociceptive pathways via the trigeminovascular system (12).

Galcanezumab, a monoclonal antibody targeting CGRP, has demonstrated efficacy in reducing headache frequency and associated symptoms in both episodic (EM) and chronic migraine (CM) (3, 13). However, its specific effects on photophobia and related functional outcomes remain insufficiently described.

This study aimed to investigate the changes in photophobia using the UPSIS-12 among patients with EM and CM receiving galcanezumab treatment. By integrating photophobia-specific measures with broader assessments of headache-related disability, mood, and anxiety, we aimed to provide a more comprehensive understanding of treatment-related improvements in both sensory and psychosocial domains.

## 2 Materials and methods

The study was designed as a retrospective observational cohort study.

### 2.1 Study population

Medical records of patients aged 18–65 years diagnosed with migraine according to the ICHD-3 were retrospectively reviewed. CM was defined based on ICHD-3 criteria as having 15 or more headache days per month, and patients with fewer than 15 headache days per month were classified as having EM. All participants were assessed by two headache specialists (EIA, PYD) following recruitment from a tertiary headache center. Patients were required to complete headache diaries for 1 month prior to study enrollment. At baseline, patients completed standardized PROMs and provided detailed sociodemographic data. Regular monthly follow-up visits entailed repeated PROMs and the review of headache diaries to record headache frequency and other characteristics.

Patients with incomplete baseline assessments or insufficient follow-up data were excluded. Throughout the study period, no additional preventive migraine therapies, antidepressants, hypnotic agents, peripheral nerve blocks, or trigger point injections were introduced. Exclusion criteria also comprised pregnancy or lactation, inability to complete study instruments, unstable medical comorbidities, and initiation or dose adjustment of psychotropic medications within 3 months prior to study entry.

### 2.2 Galcanezumab treatment protocol

A subcutaneous injection of galcanezumab (120 mg/mL solution in a single-dose prefilled syringe) was used. Patients were given a loading dosage of 240 mg (two consecutive injections of 120 mg) at the baseline appointment. A maintenance dose of 120 mg was then administered once a month during three consecutive visits, which corresponded to month 1, month 2, and month 3.

### 2.3 Study parameters

At baseline, demographic characteristics, migraine subtype, and comorbidities were recorded. Clinical evaluation included PROMs and data derived from patients’ headache diaries.

The assessed parameters included monthly headache days (MHDs), monthly migraine days (MMDs), attack duration with and without acute medication, and the frequency of using migraine non-specific analgesic (nonsteroidal anti-inflammatory drugs (NSAIDs) and paracetamol) and specific (triptans, ergots, combined therapies). Headache intensity was evaluated using a numeric rating scale (NRS), ranging from 0 (no pain) to 10 (worst pain imaginable).

PROMs included the UPSIS-12, the 12-item Allodynia Symptom Checklist (ASC-12), the Beck Depression Inventory (BDI), the Beck Anxiety Inventory (BAI), the Headache Impact Test-6 (HIT-6), and the Migraine Disability Assessment Scale (MIDAS).

Sensory symptoms, such as ictal and interictal photophobia, phonophobia, and osmophobia were assessed with a Likert scale, with scores ranging from 1 (no sensitivity) to 5 (very strong sensitivity).

Individuals achieving ≥50% reduction in MHDs from baseline to month 3 were classified as galcanezumab responders, whereas those with <50% reduction over the same period were designated as non-responders.

#### 2.3.1 Utah photophobia symptom impact scale

The UPSIS-12 is a validated instrument designed to assess the impact of photophobia on daily functioning, during and particularly between migraine episodes and the Turkish-adapted version, was used in this study (5, 6). The tool examines the impact of light exposure on daily activities, including driving, functioning in various work environments (both indoors and outdoors), and use of technological devices with screens. In addition to evaluating the intensity of photic sensitivity, the scale explains in detail whether light triggers headache onset.

UPSIS-12 consists of 12 items. Respondents to the first 10 questions rate the degree of impairment caused by photophobia on a 6-point Likert scale from 0 to 5. Items 11A-B and 12A-D provide two or three categorical response options.

This tool was intentionally designed to evaluate interictal photophobia, as laboratory-based light-sensitivity testing in the study was performed exclusively during headache-free periods. Moreover, prior tools primarily measured ictal photophobia, so the UPSIS-12 fills an unmet need by identifying the often underrecognized impact of interictal light sensitivity on daily functioning.

#### 2.3.2 Allodynia symptom checklist

The ASC-12 is a validated instrument developed to quantify cutaneous allodynia associated with migraine. The scale evaluates the presence and severity of allodynia using 12 symptom-based items, such as pain or discomfort from wearing glasses, brushing the hair, resting the head on a pillow, or shaving during a migraine attack (14). The Turkish version of the ASC-12, which has shown high reliability and validity, was used in this study (15).

#### 2.3.3 Beck depression inventory (BDI)

The BDI is a 21-item self-report tool designed to evaluate the severity of depressive symptoms during the prior 2 weeks. Each item is scored from 0 to 3, generating a cumulative score of 0 to 63. Total scores are categorized as minimal (0–9), mild (10–18), moderate (19–29), or severe depression (≥30) (16, 17).

#### 2.3.4 Beck anxiety inventory (BAI)

The BAI is a 21-item instrument that measures the presence and severity of anxiety symptoms. The items are scored from 0 (“not at all”) to 3 (“severely”), yielding a total score between 0 and 63. The levels of severity are classified as low (0–21), moderate (22–35), and severe (≥36) anxiety (18, 19).

#### 2.3.5 Headache impact test-6

The HIT-6 measures the overall impact of headache on daily function, including pain severity, social relationships, role functioning, vitality, cognitive functioning, and emotional distress. The total score ranges from 36 to 78, and every response is scored on a weighted 5-point Likert scale (never = 6 points, rarely = 8 points, sometimes = 10 points, very often = 11 points, always = 13 points). Higher scores indicate greater headache-related disability and pain burden (20, 21).

#### 2.3.6 Migraine disability assessment scale (MIDAS)

The MIDAS is a self-administered questionnaire consisting of five items that measures the level of disability resulting from migraine attacks during the preceding 3 months. It determines the number of days of lost productivity in social, domestic, and professional activities. Disability is categorized as mild (6–10), moderate (11–20), severe (≥21), and little to none (0–5) (22, 23).

#### 2.3.7 Safety outcome

Safety outcomes were evaluated by examining treatment-emergent adverse events, serious adverse events, deaths, treatment discontinuations, and changes in vital signs, including blood pressure, pulse, temperature, and body weight.

### 2.4 Statistical analysis

Statistical analyses were performed using IBM SPSS Statistics version 23. Data distribution was assessed using the Shapiro–Wilk test. For comparisons between two independent groups, the Independent Samples *t-*test was used for normally distributed variables, while the Mann–Whitney U test was applied for non-normally distributed variables. For repeated measures involving ordinal or non-normally distributed data, the Friedman test was used, followed by Wilcoxon signed-rank tests with Holm correction for *post hoc* pairwise comparisons when appropriate. Descriptive statistics were presented as mean ± standard deviation or median (minimum–maximum), as appropriate, and categorical variables were expressed as frequency (percentage).

Associations between changes in photophobia severity and migraine-related disability were evaluated using regression and correlation analyses. Changes in UPSIS-12 scores were treated as the dependent variable. Univariable linear regression included changes in HIT-6 scores as the independent variable, while Spearman’s correlation assessed the relationship between changes in UPSIS-12 and HIT-6 scores. A multivariable linear regression model incorporating changes in HIT-6, MIDAS, and cutaneous allodynia scores was used to evaluate their independent associations with changes in UPSIS-12 scores. A two-sided *p*-value < 0.05 was considered statistically significant.

## 3 Results

### 3.1 Demographic and clinical characteristics

**Figure 1:**
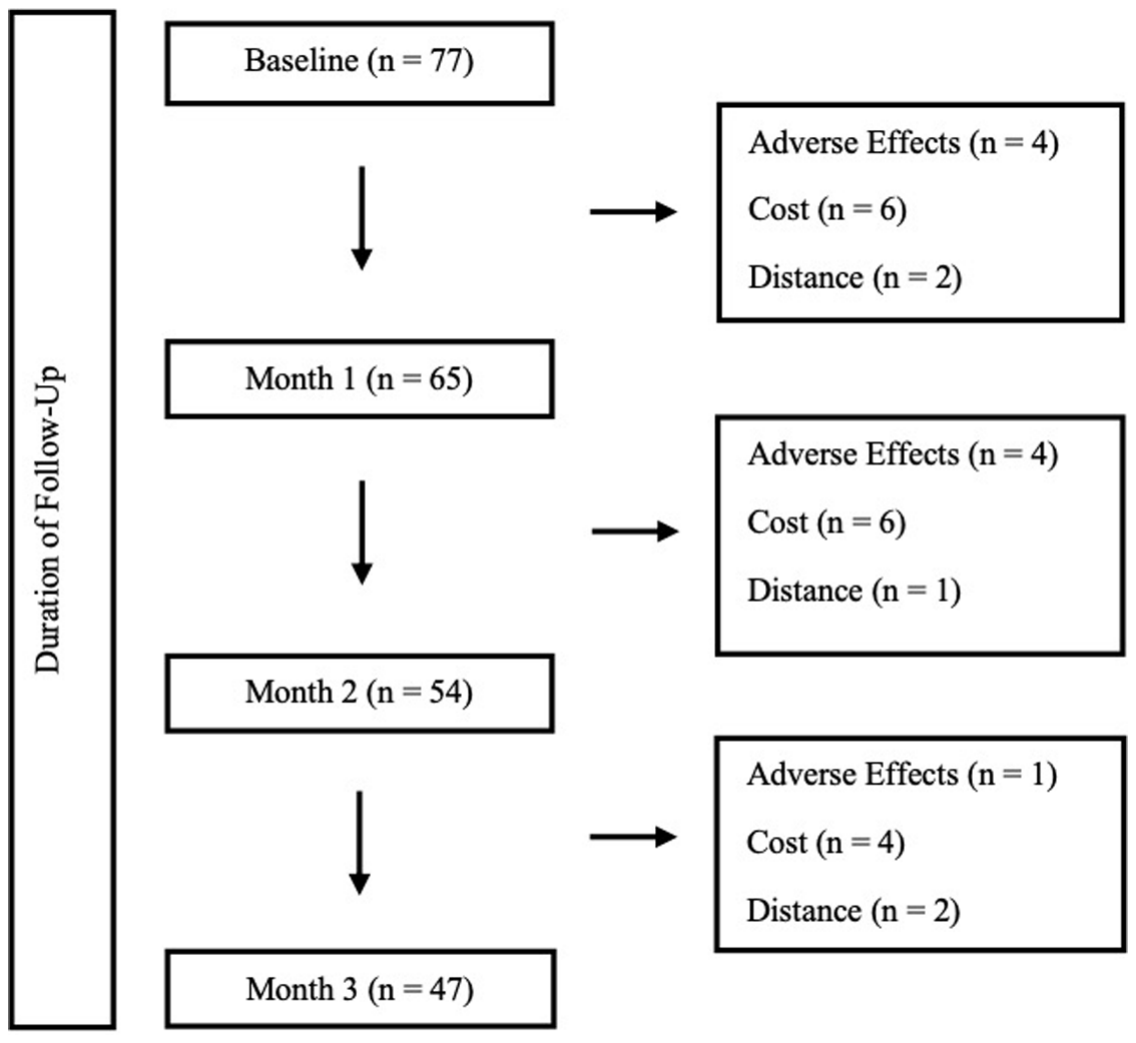
Flowchart of the participant enrollment.

A total of 77 patients were enrolled in the study; of whom 47 completed all four visits and were included in the final analysis (Figure 1). Among these 47 patients, 42 were female (89.3%). The mean age of the participants was 39.5 ± 10.6 years, and the mean body mass index (BMI) was 24.8 ± 4.6.

The patients’ educational backgrounds were distributed as follows: 46 (97.9%) had a university-level education, while 1 (2.1%) had completed high school. A family history of migraine was reported by 25 participants (53.2%). The most common comorbidities were bruxism 13 (27.7), depression in 11 (23.4%), anxiety in 10 (21.3%), sleep disorders in 10 (21.3%), and gastrointestinal complaints in 7 participants (14.9%). Eye conditions that may predispose to photophobia, such as dry eye, glaucoma, inflammatory ocular diseases, and retinal disorders, were documented in 13 patients (27.7%) (Table 1).

**Table 1:** Demographic and clinical characteristics.

Variables *n* = 47	Value
Age (year), mean (SD)	39.5 (10.6)
Gender, *n* (%)	
Female	42 (89.3)
Body mass index (kg/m^2^), mean (SD)	24.8 (4.6)
Educational status, *n* (%)	
University	46 (97.9)
High school	1 (2.1)
Primary school	–
Employment, *n* (%)	35 (74.5)
Family history for migraine, *n* (%)	25 (53.2)
Comorbid diseases, *n* (%)	
Bruxism	13 (27.7)
Depression	11 (23.4)
Anxiety	10 (21.3)
Sleep disorders	10 (21.3)
Cardiovascular disease	10 (21.3)
Gastrointestinal complaints	7 (14.9)
Ophthalmologic disorders	13 (27.7)

SD: standard deviation.

Of the 47 patients, 7 (14.9%) had migraine with aura und 32 (68.1%) met the diagnostic criteria for CM. Among these patients, 17 (36.2%) also had medication overuse headache.

### 3.2 Migraine outcomes

The amount of MHDs decreased substantially, from a median of 15 days (4–30) at baseline to 5 days (1–30) in month 1 and 2, and further to 2 days (0–30) in month 3 (*p* < 0.001) (Table 2). The median reduction by the final visit was 73.33% (mean change: 64.09% ± 27.35%).

**Table 2:** Comparison of parameters across time points.

Variable *n* = 47	Baseline	Month 1	Month 2	Month 3	Test statistics	*p*
Monthly headache days	15 (4: 30)^a^	5 (1: 30)^b^	5 (0: 30)^b^	4 (0: 30)^b^	82.601	**<0**.**001**^x^
Monthly migraine days	8 (3: 30)^a^	2 (0: 10)^b^	2 (0: 11)^b^	2 (0: 15)^b^	73.679	**<0**.**001**^x^
Attack duration with acute medication (hrs)	4 (1: 48)^a^	2 (1: 24)^b^	2 (0: 24)^b^	2 (0: 24)^b^	26.302	**<0**.**001**^x^
Attack duration without acute medication (hrs)	24 (3: 72)^a^	10 (0: 72)^b^	7 (0: 72)^b^	5 (0: 48)^b^	43.388	**<0**.**001**^x^
Headache intensity	8 (6: 10)^a^	5 (2: 10)^b^	6 (0: 10)^b^	6 (0: 10)^b^	59.854	**<0**.**001**^x^
Ictal photophobia	4 (1: 5) / 3.2^a^	3 (1: 5) /2.27^b^	3 (0: 5) / 2.05^b^	4 (1: 5) /2.48^b^	29.067	**<0**.**001**^x^
Interictal photophobia	2 (1: 5)	1 (1: 5)	1 (0: 5)	2 (1: 4)	2.031	0.566^x^
Ictal phonophobia	4 (1: 5)^a^	3 (0: 5)^b^	3 (1: 5)^b^	3 (0: 5)^b^	28.469	**<0**.**001**^x^
Interictal phonophobia	2 (1: 5)	1 (0: 5)	1 (1: 5)	1 (0: 4)	8.172	**0**.**043**^x^
Ictal osmophobia	3 (1: 5)^a^	2 (0: 5)^b^	1 (1: 5)^b^	1 (0: 5)^b^	20.375	**<0**.**001**^x^
Interictal osmophobia	2 (1: 5)	1 (0: 5)	1 (0: 11)	1 (1: 5)	15.529	**0**.**001**^x^
Migraine non-specific analgesics(days/month)	7 (0: 30)^a^	2 (0: 30)^b^	2 (0: 30)^b^	1 (0: 30)^b^	31.500	**<0**.**001**^x^
Migraine specific analgesics(days/month)	5 (0: 30)^a^	0 (0: 8)^b^	0 (0: 7)^b^	0 (0: 7)^b^	57.782	**<0**.**001**^x^
MIDAS	25 (6: 165)^a^	4 (0: 30)^b^	3 (0: 32)^b^	3 (0: 63)^b^	67.385	**<0**.**001**^x^
HIT6	66.47 ± 4.86^a^	53.7 ± 8.76^b^	52.94 ± 8.52^b^	54.87 ± 8.93^b^	55.449	**<0**.**001**^**z**^
BDI	9 (0: 40)^a^	4 (0: 38)^ab^	3 (0: 38)^b^	1 (0: 34)^b^	30.087	**<0**.**001**^x^
BAI	12 (0: 70)^a^	8 (0: 63)^b^	6 (0: 63)^b^	4 (0: 62)^b^	42.310	**<0**.**001**^x^
ASC-12	4 (0: 15)^a^	0 (0: 14)^b^	0 (0: 12)^b^	0 (0: 12)^b^	25.637	**<0**.**001**^x^
UPSIS-12	25.7 ± 11.55^a^	22.26 ± 12.21^b^	20.53 ± 13.01^b^	20.43 ± 12.41^b^	10.723	**<0**.**001**^**z**^

^x^Friedman Test; ^y^Cochran’s *Q* test; ^z^ Repeated measures analysis of variance; ^a-b^ No difference between time points sharing the same letter; Median (Minimum: Maximum); Mean ± Standard Deviation; Frequency (Percentage). ASC-12: Allodynia Symptom Checklist, BDI: Beck Depression Inventory, BAI: Beck Anxiety Inventory, HIT-6: Headache Impact Test-6, MIDAS: Migraine Disability Assessment Scale, UPSIS-12: Utah Photophobia Symptom Impact Scale. Photophobia, phonophobia, and osmophobia were graded on a 5-point Likert scale (1 = no sensitivity; 5 = very strong sensitivity). Bold values indicate statistically significant results (*p* < 0.05).

The number of MMDs decreased from a median of 8 (3–30) at baseline to 2 days at month 1 (0–10), month 2 2 (0–11), and month 3 2 (0–15), respectively (*p* < 0.001), corresponding to a median reduction of 80% over the study period.

Based on changes in MHDs, 74.5% of patients were classified as responders, defined as achieving ≥ 50% reduction in MHDs, while 25.5% were non-responders.

Headache intensity improved significantly over time (*p* < 0.001), declining from a baseline median of a NRS of 8 (6–10) to 5 (2–10) in month 1 and 6 (0–10) in month 2 and 3. The NRS declined until month 2 and remained stable between months 2 and 3 (χ^2^ = 62.191, *p* < 0.001). The mean percentage reduction in headache intensity was 31.71% ± 27.67%.

The duration of treated headache attacks with rescue medication decreased from a median of 4 h (1–48) at baseline to 2 h (1–24) across all follow-up visits (*p* < 0.001). Untreated headache attack duration decreased from 24 h (3–72) at baseline to 10 h (0–72) in month 1, 7 h (0–72) in month 2, and 5 h (0–48) in month 3 (*p* < 0.001).

The use of migraine-specific and non-specific analgesics decreased dramatically over the subsequent visits (χ^2^ = 57.782, *p* < 0.001). The number of migraine-non-specific analgesics decreased from a median of 7 days (0–30) at baseline to 2 days (0–30) in months 1 and 2, and further to 1 day in month 3 (0–30) (*p* < 0.001). Migraine-specific analgesic use showed an even more pronounced change, dropping from a median of 5 days (0–30) at baseline to 0 at all follow-up visits (*p* < 0.001).

### 3.3 Photophobia outcomes

UPSIS-12 scores decreased significantly over time across the four visits. Given the ordinal structure of the UPSIS-12 and the preference for median-based reporting, non-parametric analyses were applied.

Median UPSIS-12 scores decreased from 25.7 at baseline to 22.26 at month 1, 20.53 at month 2, and 20.43 at month 3 (*p* < 0.001). The median percentage improvement from baseline to month 3 was 22.58%, while the mean percentage change of 22.06% ± 34.5% (Table 2).

The Friedman test demonstrated a significant overall effect of time on UPSIS-12 scores (p < 0.001). *Post hoc* pairwise comparisons using the Wilcoxon signed-rank test with Holm correction showed significant reductions in UPSIS-12 scores from baseline to month 1 (adjusted *p* = 0.0077), month 1 to month 2 (adjusted *p* = 0.0005), and month 2 to month 3 (adjusted *p* = 0.0003). In contrast, comparisons between baseline and month 1 (adjusted *p* = 0.1043) and between month 2 and month 3 (adjusted *p* = 0.7439) were not statistically significant, indicating stabilization of photophobia severity after the initial treatment period.

A marked decrease in ictal photophobia, assessed using the Likert scale, was observed over time. Significant reductions were detected from baseline to month 1, 2, and 3 (all *p* < 0.001). A further decrease was observed between month 2 and 3 (*p* = 0.021), whereas no significant difference was found between month 1 and 2. In contrast, interictal photophobia did not show significant changes across visits.

No statistically significant differences were found between responders (defined as patients achieving a ≥ 50% reduction in MHDs) and non-responders in UPSIS-12 scores at baseline, at month 3, or in changes over time. Likewise, baseline and month 3 ictal photophobia, as well as interictal photophobia at baseline, month 3, and across time, did not differ significantly between the two groups.

In contrast, responders showed a significantly greater reduction in ictal photophobia during headache attacks from baseline to month 3 compared with non-responders (*p* = 0.010). This finding indicates that achieving a ≥ 50% reduction in MHDs is specifically associated with a clinically meaningful improvement in ictal photophobia (Table 3).

**Table 3:** Comparison of quantitative variables between responders and non-responders.

Variable	Change in monthly headache days		Total	Test Statistics	***p***
	Non-responder	Responder			
UPSIS-12 (Baseline)	21.5 (12: 48)	30 (4: 46)	28 (4: 48)	208.000	0.961^x^
UPSIS-12 (Visit 4)	22.25 ± 14.12	19.8 ± 11.92	20.43 ± 12.41	0.586	0.561^y^
*Change in UPSIS-12 score (Baseline-Visit 4)*	18.71 ± 34.68	23.21 ± 34.87	22.06 ± 34.5	–0.386	0.701^y^
Ictal photophobia (Baseline)	4 (2: 5)	5 (1: 5)	4 (1: 5)	192.500	0.646^x^
Ictal photophobia (Visit 4))	4 (2: 5)	4 (1: 5)	4 (1: 5)	135.000	0.060^x^
*Change in ictal photophobia (Baseline- Visit 4)*	0 (−50: 60)	20 (−100: 80)	0 (−100: 80)	107.500	*0.010^x^
Interictal photophobia (Baseline)	2 (1: 5)	2 (1: 4)	2 (1: 5)	195.500	0.708^x^
Interictal photophobia (Visit 4)	1.5 (1: 4)	2 (1: 4)	2 (1: 4)	203.500	0.864^x^
*Change in interictal photophobia (Baseline- Visit 4)*	0 (−200: 80)	0 (−200: 66.67)	0 (−200: 80)	206.500	0.924^x^

^x^Mann-Whitney *U* Test; ^y^Independent samples *t-*test; Median (Minimum-Maximum); Mean ± Standard Deviation. UPSIS-12: Utah Photophobia Symptom Impact Scale. Photophobia was graded on a 5-point Likert scale (1 = no light sensitivity; 5 = very strong sensitivity).*p < 0.05. Bold values indicate statistically significant results (*p* < 0.05).

### 3.4 Other sensory symptoms

Ictal photophobia during headache attacks improved significantly, with median scores decreasing from 4 (1–5) at baseline (mean rank: 3.2) to 3 (1–5) at month 1 (mean rank: 2.27) and 2 (0–5) at month 2 (mean rank: 2.05), and further to 4 (1–5) at month 3 (mean rank: 2.48) (*p* < 0.001). Cutaneous allodynia scores demonstrated near-complete resolution, decreasing from 4 (0–15) at baseline to 0 at all follow-up visits (*p* < 0.001). In contrast, interictal photophobia, phonophobia and osmophobia did not show a statistically significant change (Table 2).

### 3.5 Depression and anxiety

Depression and anxiety scores improved significantly over the study period. Median BDI scores decreased from 9 (0–40) at baseline to 4 (0–38), 3 (0–38), and 1 (0–34) across month 1, 2, and 3 (*p* < 0.001). Similarly, median BAI scores declined from 12 (0–70) at baseline to 8 (0–63), 6 (0–63), and 4 (0–62) over the same visits (*p* < 0.001). The greatest improvement occurred between baseline and the first follow-up visit, with modest changes thereafter. By month 3, the majority of patients had reached minimal levels of depression and anxiety symptoms (Table 2).

According to the BAI, the severity distribution of anxiety symptoms showed a marked improvement following galcanezumab treatment. At baseline, 72.34% of patients (*n* = 34) were classified as having low anxiety levels; this proportion increased to 91.49% (*n* = 43) by month 3.

Similarly, the BDI results indicated an overall reduction in depressive symptom severity. At baseline, 55.32% of patients (*n* = 26) exhibited minimal depressive symptoms, which rose to 78.72% (*n* = 37) at month 3.

### 3.6 Disability measures

HIT-6 scores showed a marked reduction, decreasing from a baseline mean of 66.47 ± 4.86 to 53.7 ± 8.76 in month 1, 52.94 ± 8.52 in month 2, and 54.87 ± 8.93 in month 3 (*p* < 0.001). All comparisons between baseline and follow-up visits were statistically significant (all *p* < 0.001).

In univariable linear regression analysis, changes in HIT-6 scores were significantly associated with changes in UPSIS-12 scores (*β* = 0.38, *p* = 0.004), explaining 17% of the variance in UPSIS-12 change (*R*^2^ = 0.17). Consistently, Spearman’s correlation analysis demonstrated a moderate positive correlation between changes in HIT-6 and UPSIS-12 scores (*ρ* = 0.39, *p* = 0.007). In the multivariable linear regression model including changes in HIT-6, MIDAS, and cutaneous allodynia scores, the overall model was statistically significant (*F* = 3.09; *p* = 0.037) and accounted for 17.7% of the variance in UPSIS-12 score changes (adjusted *R*^2^ = 0.12). Within this model, only changes in HIT-6 scores remained independently associated with changes in UPSIS-12 scores (*β* = 0.35; 95% CI = 0.07–0.64; *p* = 0.017), whereas changes in MIDAS and cutaneous allodynia scores were not significantly associated (Table 4).

**Table 4:** Multivariable linear regression analysis of change in UPSIS-12 score.

Variable *n* = 47	Beta	SE	95% CI		t	p
			LL	UL		
HIT-6	0.355	0.142	0.068	0.642	2.49	0.017*
MIDAS	0.006	0.042	−0.080	0.091	0.13	0.896
ASC-12	0.166	0.278	−0.395	0.727	0.6	0.553
Intercept	0.644	1.873	−3.135	4.422	0.34	0.733

Beta: unstandardized regression coefficient; CI: confidence interval; LL: lower limit; UL: upper limit; SE: standard error. Model statistics: *R*^2^ = 0.177; adjusted *R*^2^ = 0.120; *F* statistic = 3.09; *n* = 47. ASC-12: Allodynia Symptom Checklist, HIT-6: Headache Impact Test–6, UPSIS-12: Utah Photophobia Symptom Impact Scale–12; MIDAS: Migraine Disability Assessment Scale; *p < 0.05. Bold values indicate statistically significant results (*p* < 0.05).

### 3.7 Safety and tolerability

During the study period, approximately 32% of patients (*n* = 15) reported at least one treatment-emergent adverse event following galcanezumab administration. The most common reported adverse events were constipation (*n* = 12, 25.5%), mild injection-related reactions (*n* = 3, 6.3%), nausea (*n* = 1, 2.1%), and other (*n* = 2, 4.2%). No serious adverse events were observed, and none of the reported adverse events led to treatment discontinuation.

Following the first administration, 4 patients discontinued treatment due to injection site adverse events such as pain, erythema, and swelling, while 3 patients reported constipation but continued treatment at that stage. After the second administration, 2 patients with constipation and 2 patients with hair loss discontinued treatment. At the third visit, one additional patient who had experienced constipation since the first administration discontinued treatment due to intolerable symptoms. In total, 9 patients discontinued galcanezumab due to adverse events.

## 4 Discussion

This study aimed to explore the relationship between migraine-related sensory hypersensitivity, particularly photophobia assessed by UPSIS-12, and treatment response to galcanezumab in a real-world setting. Our findings demonstrate that while baseline UPSIS-12 scores and overall photophobia measures did not differ between responders and non-responders, a clinically meaningful reduction in monthly headache days (≥50%) was specifically associated with a significant decrease in ictal photophobia during headache attacks. This result highlights ictal photophobia as a dynamic and treatment-responsive symptom that improves alongside reductions in migraine burden. Given the prominent role of photophobia as the most MBS in migraine, these findings indicate that galcanezumab improves not only headache frequency but also light sensitivity, highlighting photophobia as an important treatment outcome in migraine care.

Galcanezumab treatment resulted also in notable improvements across multiple migraine-related domains, including headache frequency, attack severity and duration, disability, acute medication use, and associated sensory and affective symptoms. Based on changes in MHDs, 74.5% patients in our cohort were responders to galcanezumab treatment. The most pronounced benefits were observed within the first month of treatment, followed by stabilization over subsequent visits, indicating a rapid onset of therapeutic effect with sustained efficacy. A notable finding was the marked reduction in both migraine-specific and non-specific acute medication use, with migraine-specific analgesics declining to near zero, with important implications for reducing medication overuse headache.

Overall, galcanezumab treatment was associated with a 22–23% improvement in photophobia severity measured by the UPSIS-12, while Likert scales indicated a 35–40% reduction in attack-related photophobia. However, patients declared no improvement in attack-free photophobia with the Likert scale, underscoring the necessity of comprehensive instruments such as UPSIS-12 for accurately evaluating photophobia outcomes. The UPSIS-12 is specifically designed to assess both ictal and interictal photophobia, with particular emphasis on a detailed set of items that address light intolerance during headache-free periods. Our findings illustrate that single-item Likert ratings cannot fully capture photophobia severity, as patient-reported assessments may fail to reflect the actual burden of persistent light sensitivity outside migraine attacks. Interictal photophobia represents a clinically important but often underestimated component of migraine burden. Previous studies have shown that photophobia between attacks is independently associated with reduced quality of life, functional impairment, and higher psychiatric symptom burden, even in the absence of headache (24, 25). The Türkiye real-world study of galcanezumab in migraine treatment showed that 10.7% of patients reported no photophobia at baseline, while 17.6% experienced no improvement with treatment. In contrast, a substantial proportion demonstrated marked benefit, with 34.9% achieving a 50–99% reduction and 22.3% reporting complete resolution of photophobia during migraine attacks (3).

The significant improvement in ictal photophobia, phonophobia, osmophobia, and the near-complete resolution of cutaneous allodynia observed in our cohort reflect a substantial reduction in attack-related sensory hypersensitivity following galcanezumab treatment, in line with prior experimental and clinical findings in migraine (26). Furthermore, responders exhibited a significantly greater reduction in ictal photophobia from baseline to Month 3 compared with non-responders (*p* = 0.010), suggesting that achieving a ≥ 50% reduction in MHDs is associated with a clinically meaningful attenuation of attack-related light sensitivity.

From a pathophysiological perspective, CGRP monoclonal antibodies are thought to attenuate ictal photophobia by modulating a CGRP-sensitive pathway that conveys light signals from melanopsin-containing intrinsically photosensitive retinal ganglion cells to the posterior thalamus and, subsequently, to trigeminovascular pain networks (27). Experimental models have demonstrated that CGRP signaling contributes to light aversion (28). In contrast, interictal photophobia appears to be linked to more stable cortical hyperexcitability, and altered thalamocortical processing, as show on interictally on functional imaging, and may be less responsive to short-term CGRP inhibition (29, 30).

Our findings are in line with prior studies showing that photophobia improves with migraine preventive treatments. Schiano di Cola et al. (31) reported relief of ictal photophobia in nearly two-thirds of patients with episodic and chronic migraine treated with galcanezumab. Extending these observations, our study used the validated UPSIS-12 scale to assess light sensitivity, allowing a more comprehensive evaluation of both ictal and interictal photophobia.

Previous clinical studies have also reported reductions in photophobia with other preventive therapies, including topiramate, amitriptyline, and onabotulinumtoxin A, supporting photophobia as a modifiable symptom rather than a fixed trait (32, 33). Patients responding to anti-CGRP monoclonal antibodies such as erenumab, galcanezumab, or fremanezumab demonstrated significant reductions in photophobia, phonophobia, and aura, with greater improvements observed in those with higher baseline photophobia (34–36). In contrast, in our study, baseline UPSIS-12 scores, and levels of ictal and interictal photophobia did not significantly differ between galcanezumab responders and non-responders.

Migraine-related disability markedly decreased, with significant reductions in both HIT-6 and MIDAS scores. The rapid decline in MIDAS scores from severe to minimal disability within the first month highlights the early and clinically meaningful impact of galcanezumab on overall migraine burden as stated by previous studies (37–39). Multivariable regression and correlation analyses demonstrated that improvements in headache-related disability (HIT-6), independently predicted and were associated with reductions in photophobia severity, indicating that relief of light sensitivity accompanies functional recovery during migraine prevention.

Notably, in our cohort, the majority of patients reached minimal levels of depression and anxiety by month 3. The progressive reductions in BDI and BAI scores across flow-up visits are consistent with growing real-world data from clinical cohorts in Japan and Turkey, demonstrating that galcanezumab treatment reduces depressive and anxiety symptoms (40, 41). Moreover, a *post hoc* analysis of phase 3 trials revealed that patients with comorbid anxiety and/ or depression achieved reductions in monthly migraine days comparable to those without such comorbidities, indicating that psychiatric improvement may accompany the overall clinical benefit of migraine prophylaxis (42).

Several limitations must be acknowledged. The study was conducted over a relatively short 3-month period, which may not reflect slower or longer-term sensory adaptations. The sample size was modest, and headache frequency and associated symptoms were assessed through PROMs, potentially introducing subjective bias and recall variability even with the use of some validated instruments. In addition, due to the inclusion of a substantial proportion of patients with chronic migraine in our cohort, the assessment of interictal photophobia may have been inherently challenging, as the limited number of headache-free days in this population can restrict the accurate evaluation of sensory symptoms outside migraine attacks. The relatively high dropout rate may introduce some degree of selection bias and should be considered when interpreting the findings. As this was a retrospective study without a control or placebo group, the findings should be interpreted with caution. Subgroup analyses, including comparisons between responders and non-responders, should be considered exploratory given the limited sample size and short follow-up period, and should not be overinterpreted.

Future studies with larger cohorts, longer observation periods, and neurophysiological or imaging correlates are warranted to further elucidate the mechanisms underlying persistent interictal photophobia and its responsiveness to preventive migraine therapies.

## 5 Conclusion

In summary, galcanezumab treatment was associated with a reduction in light sensitivity, alongside marked decreases in monthly headache and migraine days, headache intensity, acute medication use, depression, and anxiety symptoms. To better characterize the nature of migraine-associated symptoms and their response to treatment, and to further advance our understanding of migraine pathophysiology, the broader adoption of validated migraine-specific instruments, such as UPSIS-12, as well as the development and systematic use of additional tools, may be beneficial. Continued research with long-term follow-up and larger, controlled studies are needed to confirm these observations and to explore how changes in sensory processing may contribute to overall improvement in migraine.

## Data Availability

The original contributions presented in the study are included in the article/supplementary material, further inquiries can be directed to the corresponding author/s.
